# Deregulated methylation and expression of *PCDHGB7* in patients with non-small cell lung cancer: a novel prognostic and immunological biomarker

**DOI:** 10.3389/fimmu.2025.1516628

**Published:** 2025-01-30

**Authors:** Yue Yuan, Xin Nie, Jiayi Gao, Yumeng Tian, Liuer He, Xue Wang, Ping Zhang, Junling Ma, Lin Li

**Affiliations:** ^1^ Department of Oncology, Beijing Hospital, National Center of Gerontology, Institute of Geriatric Medicine, Chinese Academy of Medical Sciences, Beijing, China; ^2^ Beijing Hospital, National Center of Gerontology, Institute of Geriatric Medicine, Chinese Academy of Medical Sciences & Peking Union Medical College, Beijing, China

**Keywords:** *PCDHGB7*, methylation, lung cancer, immunotherapy, biomarkers

## Abstract

**Backgrounds:**

Protocadherin gamma subfamily B, 7 (PCDHGB7), a member of the protocadherin family, plays critical roles in neuronal connections and has been implicated in female reproductive system cancers. Its function in lung cancer has not been elucidated.

**Methods:**

We comprehensively investigated PCDHGB7 expression, prognosis, biological function, methylation patterns, and it’s relationship with immune infiltration and immunotherapy response through public datasets (HPA, TCGA, GEO, OncoDB and MEXPRESS). Two lung cancer immunotherapy cohorts from our clinical center were enrolled to detect the relationship between methylation and protein levels of *PCDHGB7* in plasma and immunotherapy outcomes.

**Results:**

*PCDHGB7* expression was downregulated in lung adenocarcinoma (LUAD) and lung squamous cell carcinoma (LUSC) and associated with tumor prognosis. *PCDHGB7* demonstrated a positive correlation with inhibitory immune cells and a negative correlation with tumor mutational burden (TMB) and homologous recombination deficiency (HRD). The methylation level of *PCDHGB7* was upregulated in tumor tissue and negatively correlated with *PCDHGB7* mRNA level. In immunotherapy cohort studies, patients with higher *PCDHGB7* tissue expression showed worse prognosis. Patients with *PCDHGB7* hypermethylation in baseline plasma had shorter progression-free survival (PFS) and overall survival (OS), while those with early reduction of *PCDHGB7* methylation had the best prognosis. Plasma *PCDHGB7* protein levels could predict responses to immune checkpoint inhibitors and function as a prognostic marker for PFS.

**Conclusion:**

*PCDHGB7* expression and methylation are prognostic and immunological biomarkers in non-small cell lung cancer. Plasma *PCDHGB7* methylation and protein levels can be used as novel biomarkers for predicting the efficacy of immunotherapy in lung cancer.

## 1 Introduction

Lung cancer continues to be the leading cause of death in all demographics and a significant factor in the global decline in health-related quality of life (1). Immunotherapy has become a powerful and vital treatment approach with the introduction of immune checkpoint inhibitors (ICIs), such as anti-programmed death 1 (PD-1), anti-programmed death-ligand 1 (PD-L1), and anti-cytotoxic T lymphocyte-associated protein 4 (CTLA-4). Nonetheless, low response rates and drug resistance remain the main reasons, which hinder the efficacy of immunotherapy (2). Thus, tumor mutational burden (TMB), PD-L1, and other biomarkers have been used as indicators for predicting the effectiveness of immunotherapy (3, 4), which also played important roles in regulating the tumor microenvironment (TME) (5). However, identifying novel biomarkers for optimizing the therapeutic impact of immunotherapy is still in an urgent need since the existing indicators, such as PD-L1 and TMB, which rely on tumor tissue biopsy, still cannot meet the clinical needs due to the lack of invasive examination, inconsistent detection standards, and inability to detect dynamically.

Protocadherin gamma subfamily B, 7 (PCDHGB7) is a member of the protocadherins (PCDH) family, representing the largest group within the cadherin superfamily (6). The protein structure of PCDHGB7 comprises multiple β sheets and it is mainly localized to the vesicles and plasma membrane, with an additional presence detected in the nucleoplasm and cytosol (**Supplementary Figures S1A, B**). According to the Human Protein Atlas (HPA) database, *PCDHGB7* exhibited high RNA expression in lung and cerebral cortex, as well as ovaries, endometrium, cervix and other female reproductive systems (**Supplementary Figure S1C**). At the protein level, PCDHGB7 was extensively expressed across multiple organs, with particularly high expression observed in the cervix, ovaries, and cerebral cortex. Low protein expression was also noted in the bronchial and pulmonary tissues (**Supplementary Figure S1D**). According to previous research, PCDHGB7 is involved in synaptic mobility, self- and mutual recognition, and the formation of the nervous system network (7–9). Of note, several studies have found that PCDHGB7 contributed to cancer development and functioned as a tumor suppressor gene. It was reported that protocadherins suppressed the development of tumors via multiple biological interventions, such as promoting cell cycle arrest, inducing apoptosis, and regulating the Wnt (Wingless and INT-1) pathway (10, 11). However, the role and function of PCDHGB7 in cancers have not been fully elucidated.

DNA methylation abnormalities have been shown to precede pathological changes (12, 13). Previous studies based on TCGA and GEO databases (n=7114) have demonstrated that *PCDHGB7* was hypermethylated in all cancer types. Clinical cancer samples (n=727) detection revealed that *PCDHGB7* hypermethylation could be a diagnostic biomarker for cancer detection in a variety of cancers (14). The level of *PCDHGB7* methylation has been then proven to be an early and reliable diagnosis indicator of cervical cancer and has shown good diagnostic efficacy in the diagnosis of malignant body fluids (14–16). *PCDHGB7* has been found to upregulated in lung cancer, while the specific function of *PCDHGB7* remains to be further clarified.

In this study, we investigated the role of *PCDHGB7* in lung cancers via integration of multiple bioinformatics methods. *PCDHGB7*-associated gene expression, biological function, genomic alterations, immune infiltration, and methylations were extensively studied in our work. The *PCDHGB7* methylation and expression in plasma was also detected in lung cancer patients collected in our clinical center in order to reveal the association between *PCDHGB7* and immunotherapy response. In summary, our research aim to contribute to a better understanding of the role of *PCDHGB7* in lung cancer, and expose the potential of *PCDHGB7* to be a novel biomarker for lung cancer.

## 2 Materials and methods

### 2.1 TCGA data acquisition and preprocessing

We downloaded the standardized pan-cancer dataset of The Cancer Genome Atlas (TCGA) repository (https://www.cancer.gov/ccg/research/genome-sequencing/tcga) from, the UCSC (https://xenabrowser.net/) database (accessed on May 17, 2024). Gene expression data and clinical parameters were sorted, merged, and normalized by PERL scripts and related R packages (R version: 4.3.3).

### 2.2 Expression analysis and prognostic analysis of *PCDHGTB7*

The architectural conformation and subcellular localization (Rh30, SK-MEL-30 and U2OS cell lines) of *PCDHGTB7* were inferred from data accessible in the Human Protein Altas (HPA) repository (https://www.proteinatlas.org/). Using R package “survival” (version 4.3.3) to analyze the association between *PCDHGB7* expression and prognosis (Data with survival time or follow-up time less than 30 days were excluded). Meanwhile, we use the online tool KMplot(https://kmplot.com/analysis/) to invoke the Gene Expression Omnibus (GEO) database (GSE19188, GES157011 and GSE102287) to draw Kaplan–Meier survival plots(accessed on May 17, 2024). The median values served as the cutoff points for high and low levels of *PCDHGB7* expression in all analyses.

### 2.3 Immune infiltration and immunotherapy cohorts analysis of *PCDHGB7*

The examination of the associations between *PCDHGB7* expression and the tumor mutation burden (TMB), microsatellite instability (MSI) as well as homologous recombination deficiency (HRD) from TCGA cohorts, was conducted through the Sanger Box platform. Pearson’s rank correlation test was executed, yielding both the partial correlation (cor) and corresponding p-value.

The ESTIMATE algorithm of LUAD and LUSC was used to analyze the disparity in the stromal score and the immune score with the package “estimate” (Version R4.2.1). Explorations into the connections between *PCDHGB7* expression and tumor-infiltrating immune cells (TIICs), such as B cells, CD4+T memory cells, CD8+T cells, NK cells, monocytes, macrophages, among others was achieved through quanTIseq algorithm using package “quanTIseq” (Version R4.2.1).The immunotherapy cohorts of LUAD and LUSC were sourced from the BEST database (https://rookieutopia.com/appdirect/BEST/) (17).

### 2.4 Tumor stemness analysis of *PCDHGB7*

We obtained the methylation-based stemness score (DNAss) and epigenetically regulated DNA methylation-based stemness score (EREG-METHss) based on one-class logistic regression machine learning algorithm (OCLR) of LUAD and LUSC (18). Afterwards, we used the Pearson function in R studio to calculate the correlation between *PCDHGB7* expression and tumor stemness indicators.

### 2.5 Methylation evaluation of *PCDHGB7*

OncoDB *(* 19) (https://oncodb.org/) and MEXPRESS (20) (https://mexpress.be/) databases were used in our work to assess the *PCDHGB7* DNA methylation levels.

### 2.6 Pathway and functional enrichment analyses

We retrieved RNA-Seq data of TCGA-LUAD and TCGA-LUSC and then stratified PCDHGB7 expression into high and low expression cohorts and conducted both Kyoto Encyclopedia of Genes and Genomes (KEGG) pathway and Gene Ontology (GO) enrichment analyses on the differentially expressed genes between these two cohorts (LUAD: |logFC| > 1.5, p < 0.01, FDR < 0.01, LUSC: |logFC| > 1.0, p < 0.01, FDR < 0.01). KEGG and GO analysis were performed by the enrichGO function and enrichKEGG function in the “clusterProfiler” package of R studio, respectively. Gene set enrichment analysis (GSEA) was performed using GSEA software (version 4.1.0).

### 2.7 Detection of PCDHGB7 methylation in plasma

Patients from October 2018 to November 2022 with pathologically or immunohistochemically proven unresectable locally advanced or advanced non-small-cell lung cancer (ECOG score 0–2) in the Oncology Department of Beijing hospital were enrolled. All patients received monotherapy or a combination regimen containing PD-1inhibitors. RECIST 1.1 was used to evaluate the efficacy of solid tumors, and survival analysis was performed based on follow-up data. Peripheral blood plasma was collected before and at the early stage of treatment (after 2–4 courses of treatment), and for some patients, plasma collection occurred at several time points throughout treatment. All patients provided written informed consent. This study was approved by the ethics committee of the National Cancer Center/Cancer Hospital, Chinese Academy of Medical Sciences, and Peking Union Medical College (Beijing, China; approved no. NCC2021C-527).

For sample preprocessing, 8–10 mL of whole blood was collected in a 10-mL purple-capped EDTA anticoagulant tube. The first centrifugation was performed within 4 h at 4°C, with the blood centrifuged at 3000 g for 10 min, and the upper layer of plasma was carefully transferred to a new 5-mL EP tube. Subsequently, a second centrifugation was performed at 4°C and 16, 000 g for 10 min, and the upper layer of plasma was carefully transferred again to a new 1.5-mL EP tube.

For DNA extraction and methylation analysis, ctDNA from plasma samples (400μL) was extracted using the Tiangen Enhanced Magnetic Beads Large Volume Free DNA Extraction Kit (DP720-02). Starting with 100 ng of DNA, bisulfite conversion (Zymo, D5006) was performed, followed by two rounds of nested PCR before pyrosequencing analysis to assess *PCDHGB7* CpG methylation. DNA extraction and methylation analyses were performed by Shanghai Epiprobe Biotechnology Co., Ltd. Each sample requires 1mL of plasma for testing.

Some patients have already undergone targeted capture sequencing of 1021 genes in previous studies at our center, and PyClone was used to infer the molecular tumor burden index (mTBI) (21).

### 2.8 Detection of plasma *PCDHGB7* protein levels

This was an observative real-world study performed in patients with locally advanced or advanced non-small-cell lung cancer (NSCLC) of Beijing Hospital between May 2020 and June 2023 who progressed after a list-one-line systematic therapy. Patients aged 65 or older with an ECOG score of 0–3 assessed by one tumor specialist were eligible for inclusion. Those who tested positive for driver genes were subsequently evaluated by two oncologists to determine their candidacy for immunotherapy following standard treatment protocols. All patients received PD-1 inhibitors (200 mg IV day 1, every 3 weeks) in combination with metronomic oral vinorelbine (30 mg, 3 times per week). After six cycles of combined therapy, patients who did not experience progression or for whom treatment was paused due to intolerable adverse reactions were switched to monotherapy with PD-1 inhibitors for maintenance treatment. Peripheral plasma was collected from all patients at the beginning and early stages of treatment (1–3 cycles after treatment). All patients provided written informed consent. The present study was approved by the ethics committee of Beijing Hospital (Beijing, China; approved no. 2023BJYYEC-196-01).

Plasma samples (50 μL) were analyzed using the SOMAscan Assay Kit for human serum, which measures the expression of 11000 types of human proteins using highly selective single-stranded modified Slow Off-rate Modified DNA Aptamers (SOMAmer) according to the manufacturer’s standard protocol (SomaLogic; Boulder, CO).

### 2.9 Statistical analysis

Data processing, statistical analysis, and visualization were performed comprehensively using the R 4.3.3 software package and GraphPad Prism 10.0 software. For datasets exhibiting a normal distribution, the unpaired Student’s t-test was applied. The associations between categorical variables were analyzed using Pearson’s chi-square test. Pearson’s correlation coefficients were used to evaluate the associations between two continuous variables. Considering the potential impact of skewed data, Spearman’s correlation analysis was also performed to ensure a comprehensive examination of the relationship. The prognostic value was evaluated by Kaplan–Meier analysis. A significance level of P < 0.05 (two-tailed) was considered statistically significant, and the false discovery rate (FDR) was controlled by the Benjamini–Hochberg procedure.

## 3 Results

### 3.1 *PCDHGB7*’s expression and survival research in lung adenocarcinoma and lung squamous cell carcinoma

**Figure 1:**
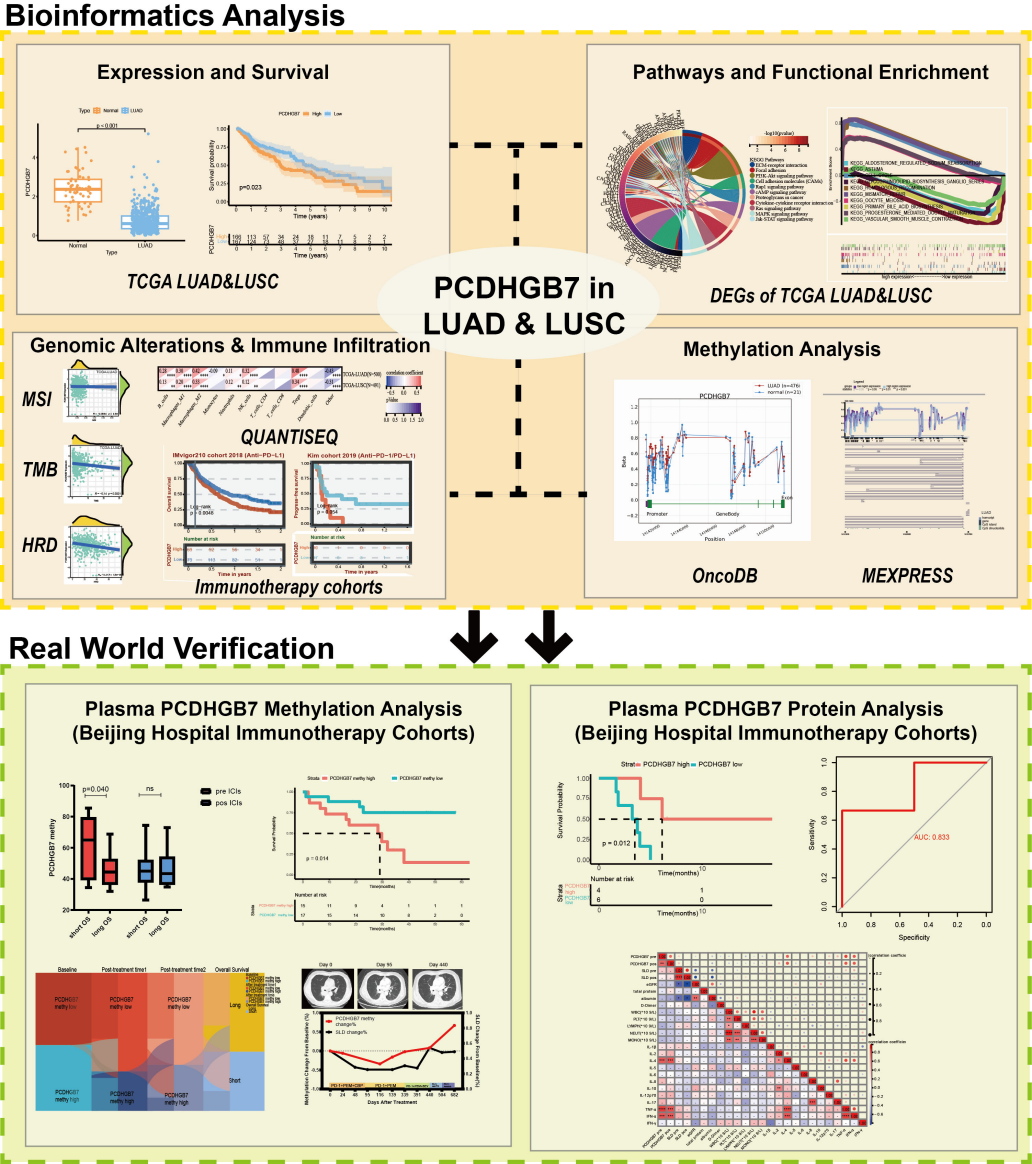
The flow chart of the study.

**Figure 2:**
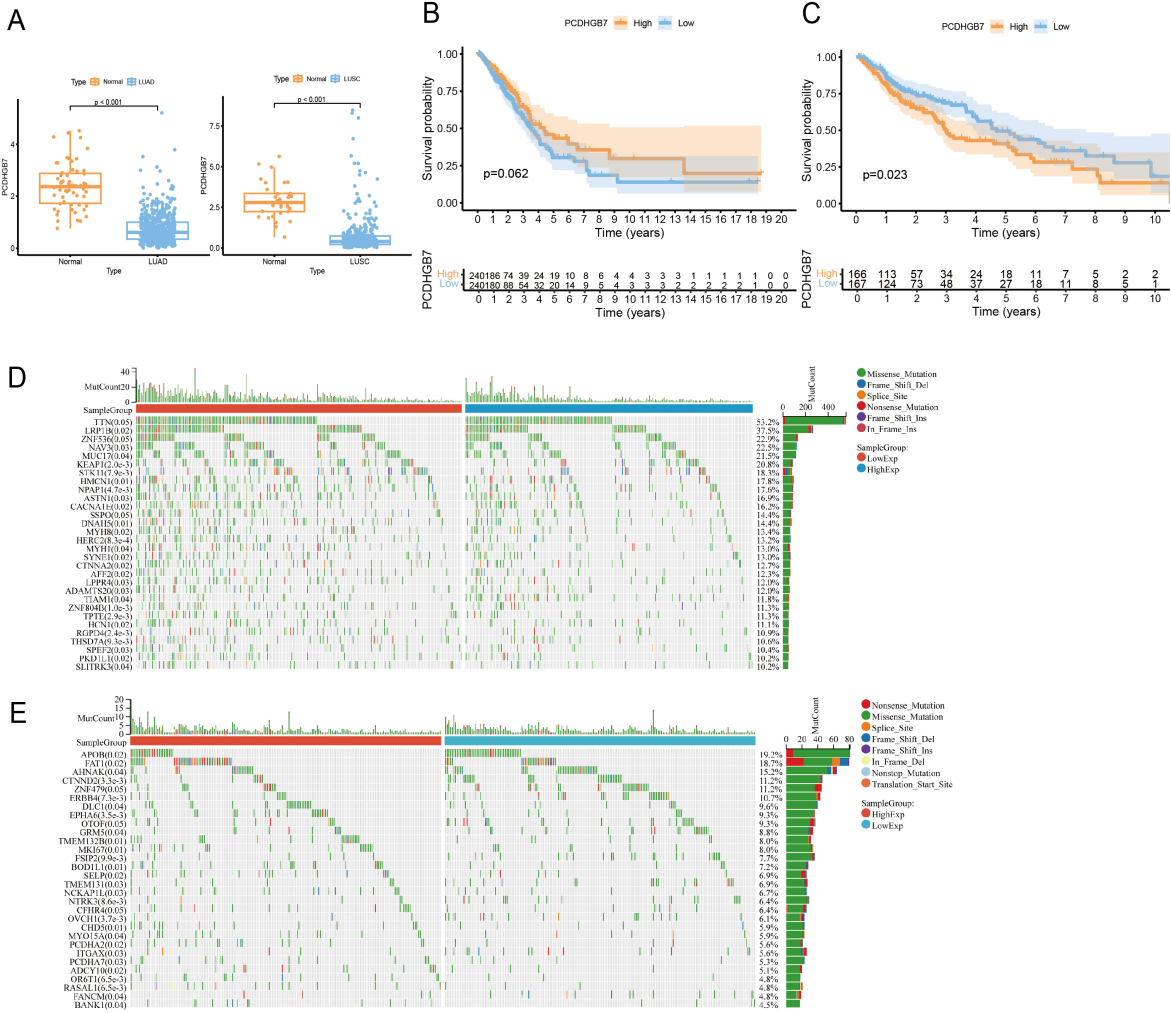
*PCDHGB7*’s expression, prognostic and mutations analysis in lung cancer. **(A)** *PCDHGB7* expression in LUAD (normal *n* = 59, tumor *n* = 541. *p <*0.0001) and LUSC (normal *n* = 34, tumor *n* = 360, *p <*0.0001). **(B)** Kaplan-Meier survival analysis of *PCDHGB7* expression in TCGA-LUAD(*p*=0.062). **(C)** Kaplan-Meier survival analysis of *PCDHGB7* expression in TCGA-LUSC(*p*=0.023). Mutation landscape of LUAD **(D)** and LUSC **(E)**.

The study’s procedural overview is depicted in **Figure 1**. Given the high expression of *PCDHGB7* within pulmonary tissue, we aimed to elucidate the role of *PCDHGB7* in lung cancer. *PCDHGB7* showed significantly low expression in both LUAD (p <0.0001) and LUSC (p <0.0001) (**Figure 2A**). Next, we used R script to examine the prognostic relevance of *PCDHGB7* expression in lung cancer. **Figure 2B** showed that low *PCDHGB7* mRNA expression showed a trend of poor overall survival (OS) in patients with LUAD (p=0.062), while low *PCDHGB7* mRNA expression was significantly associated with better OS in patients with LUSC(p=0.023) (**Figure 2C**). Due to the contradictory results of prognostic analysis, we further used the KMplot online resource to extract data from the Gene Expression Omnibus (GEO) database. The results of the GSE19188, GES157011 and GSE102287 datasets indicated that patients with high *PCDHGB7* expression exhibited poor OS compared with those with low expression levels (**Supplementary Figure S2**). These results suggest that high *PCDHGB7* expression could lead to a poor prognosis in patients with lung cancer.

### 3.2 *PCDHGB7*-associated mutation landscape in lung cancer

As gene mutations play an important role in lung cancer development, we next characterized the genomic mutation profile correlated with *PCDHGB7* expression in lung cancer. *PCDHGB7* expression in LUAD was associated with gene mutations such as *LRP1B*, *STK11*, and *KEAP1*. Of note, these genes mutations have been shown to have predictive effects on immune therapy for lung cancer in previous studies (**Figure 2D**) (4, 22). Meanwhile, patients with high expression of *PCDHGB7* in LUSC were more likely to develop FAT1 mutations, which would contribute to tumor development (**Figure 2E**) (23).

### 3.3 *PCDHGB7*-related pathways and functional enrichment analysis in LUAD and LUSC

**Figure 3:**
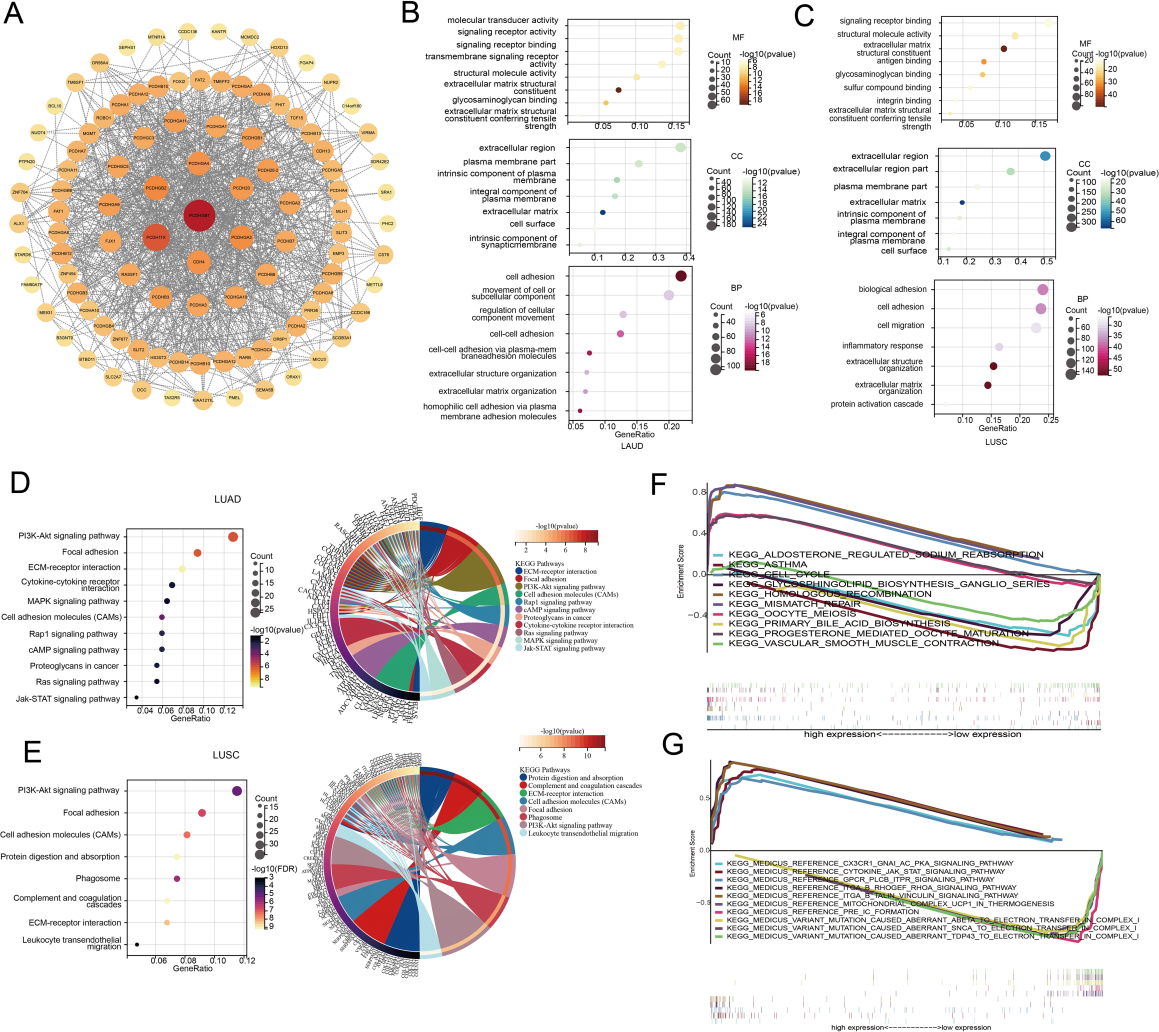
Pathway and functional enrichment analyses of LUAD and LUSC. **(A)** Protein interaction network of *PCDHGB7* (constructed and visualized using Cytoscape software). **(B)** GO of LUAD. **(C)** GO of LUSC. **(D)** KEGG of LUAD. **(E)** KEGG of LUSC. **(F)** GSEA of LUAD. **(G)** GSEA of LUSC.

To further explore the biological function of *PCDHGB7*, we retrieved and downloaded the proteins closely interacting with *PCDHGB7* from the STRING database and visualized the protein interaction network using Cytoscape. In addition to other members of the PCDH family, PCDHGB7 is closely related to proteins such as Protocadherin Fat 1(FAT1), mutL homolog 1(MLH1), and fragile histidine triad diadenosine triphosphatase (FHIT), which have been shown to be involved in DNA mismatch repair and related to tumor stemness (**Figure 3A**) (23, 24). Collectively, these data suggested that high *PCDHGB7* expression may promote a hybrid EMT state due to dysfunction of FAT1 protein, tumor stemness, and metastasis.

To investigate the function of *PCDHGB7* in lung cancer with greater precision, we conducted GO and KEGG pathway enrichment analyses based on the differentially expressed genes between the *PCDHGB7*-high and *PCDHGB7-*low groups. The results of the GO analysis indicated that *PCDHGB7* primarily contributes to the cellular component by being a part of the cell membrane, the intrinsic component of the plasma membrane, and the extracellular matrix. In terms of molecular function, *PCDHGB7* is mainly involved in signal receptor binding and activity, as well as maintaining the structural integrity of the extracellular matrix. Regarding biological processes, *PCDHGB7* predominantly participates in cell adhesion, homophilic cell-cell adhesion, and the organization of the extracellular matrix. (**Figures 3B, C**). KEGG analysis showed that *PCDHGB7* was associated with typical tumor proliferation pathways including phosphatidylinositol 3’-kinase (PI3K)-Akt signaling pathway, mitogen-activated protein kinase (MAPK) signaling pathway, Janus kinase/signal transducers, and activators of transcription (JAK-stat) signaling pathway. *PCDHGB7* also participated in leukocyte transactional migration, cytokine receptor interaction and other immune response processes (**Figures 3D, E**). GSEA revealed that the high expression of *PCDHGB7* was associated with the upregulation of homologous recombination, mismatch repair, and cell cycle pathways in LUAD (**Figure 3F**), as well as the upregulation of the JAK-stat pathway and CX3CR1-GNA-AC-PKA signaling pathway in LUSCs (**Figure 3G**). The findings mentioned earlier indicated that *PCDHGB7* is crucial for cell communication, tissue structure, and potentially for processes such as cell migration and differentiation. Additionally, *PCDHGB7* plays a significant role in DNA homologous recombination and mismatch repair within lung cancer. It also contributes to pathways that are central to tumor proliferation. The involvement of *PCDHGB7* in these critical biological processes suggests that it could be a pivotal factor in both the development and progression of lung cancer.

### 3.4 Genomic alterations of *PCDHGB7* in LUAD and LUSC

**Figure 4:**
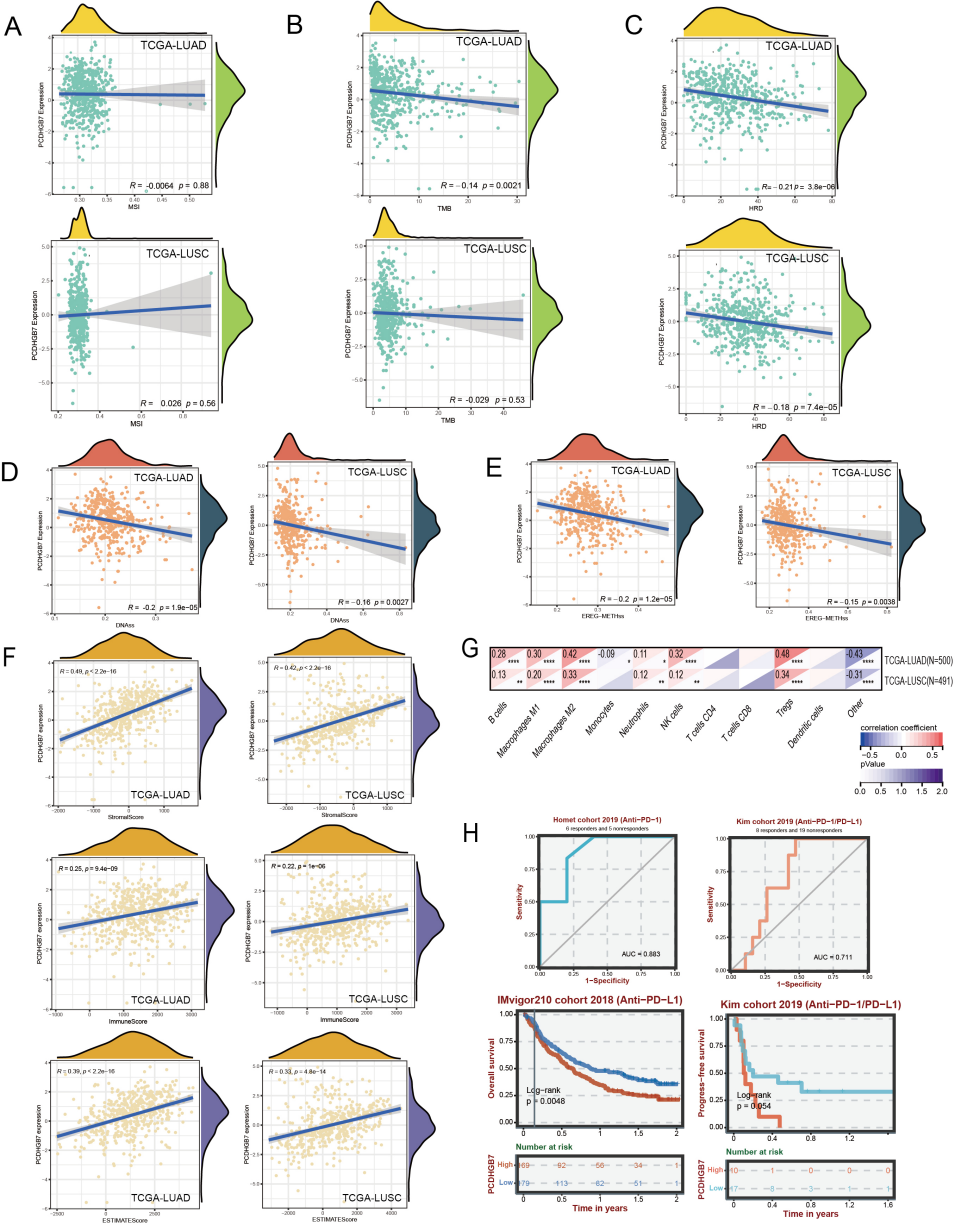
Genomic alterations and immune assessment of *PCDHGB7* in lung cancer. **(A)** Correlation with microsatellite. **(B)** Correlation with tumor mutation burden. **(C)** Correlation withHRD. Correlation with homologous recombination repair defects: **(D)** DNAss score. **(E)** EREG-METHss score. **(F)** ESTIMATE scores of LUAD and LUSC. **(G)** Correlation of PCDHGB7 expression levels with immune cell infiltration based on quanTIseq (*P < 0.05, **P < 0.01, ***P < 0.001, ****P < 0.0001). **(H)** ROC curves and Kaplan-Meier survival analysis in the immunotherapy cohort analysis.

The association between *PCDHGB7* and biomarkers related to genomic alterations such MSI, TMB, and HRD was then assessed. There was no statistically significant connection between *PCDHGB7* expression and MSI in either LUAD (r = -0.0064, *p* = 0.88) or LUSC (r = 0.026, *p* = 0.56) (**Figure 4A**). In the TMB analysis, *PCDHGB7* expression showed a significant negative correlation with TMB in LUAD (r = -0.14, *p* = 0.0021), but no significant correlation was seen in LUSC (r = -0.029, *p* = 0.53) (**Figure 4B**). In contrast, *PCDHGB7* expression showed a significant correlation with HRD, both in LUAD (r = -0.21, *p<*0.001) and LUSC (r = -0.18, *p<*0.001) (**Figure 4C**).

Cancer progression is mediated by the gradual loss of a differentiated phenotype and the acquisition of progenitor and stem cell-like features. Here, we used two types of one-class logistic regression machine learning algorithms to assess the degree of oncogenic dedifferentiation based on previously published results (18). The DNAss score (**Figure 4D**) and EREG-METHss score (**Figure 4E**) demonstrated that *PCDHGB7* expression was negatively correlated with tumor stemness LUAD (r = -0.2, *p<*0.001) and LUSC (r = -0.16, *p=*0.0027). Empirical evidence from prior research has indicated that the stemness characteristics of neoplasms were correlated with tumor grade, processes of invasion and metastasis, as well as the reduction in PD-L1 expression has been observed (18). Consequently, these data suggest that *PCDHGB7*play a significant role in the pathobiological progression of tumor development.

### 3.5 Immune infiltration and real-world cohort immune response analysis of *PCDHGB7*

The TME fundamentally affects the progression of cancer, which is a complex of tumor cells, stromal elements, and immune components, the TME orchestrates intricate and dynamic interactions (25). The ESTIMATE algorithm has emerged as a robust computational tool for quantifying the infiltration of stromal and immune cells into tumor by calculating immune scores and stromal scores. The immune scores showed that *PCDHGB7* expression was slightly positive correlated with immune infiltration in LUAD and LUSC (**Figure 4F**). Then we used mRNA-based immune infiltration prediction algorithm quanTIseq to further explore immune cell infiltration. The analysis of LUAD and LUSC both showed a significant positive correlation between *PCDHGB7* and M2 macrophages (LUAD: r=0.42, P < 0.0001; LUSC: r=0.33, P < 0.0001) as well as Treg cells (LUAD: r=0.48, P < 0.0001; LUSC: r=0.34, P < 0.0001) compared to other immune cell subgroups (**Figure 4G**).

Analysis of mutation landscape and immune infiltration suggested that *PCDHGB7* expression is related to the efficacy of lung cancer immunotherapy. To further decipher the effect of *PCDHGB7* in real-world setting in patients with lung cancer undergoing immunotherapy, the BEST database was employed, and the data from the IMvigor210 cohort and Kim cohort showed that *PCDHGB7* expression functioned as an effective predictor for immunotherapy response (**Figure 4H** AUC curves). Patients with high expression of *PCDHGB7* exhibited significantly poor responses to immunotherapy (**Figure 4H** survival curves).

### 3.6 Methylation analysis of *PCDHGB7* in LUAD and LUSC

**Figure 5:**
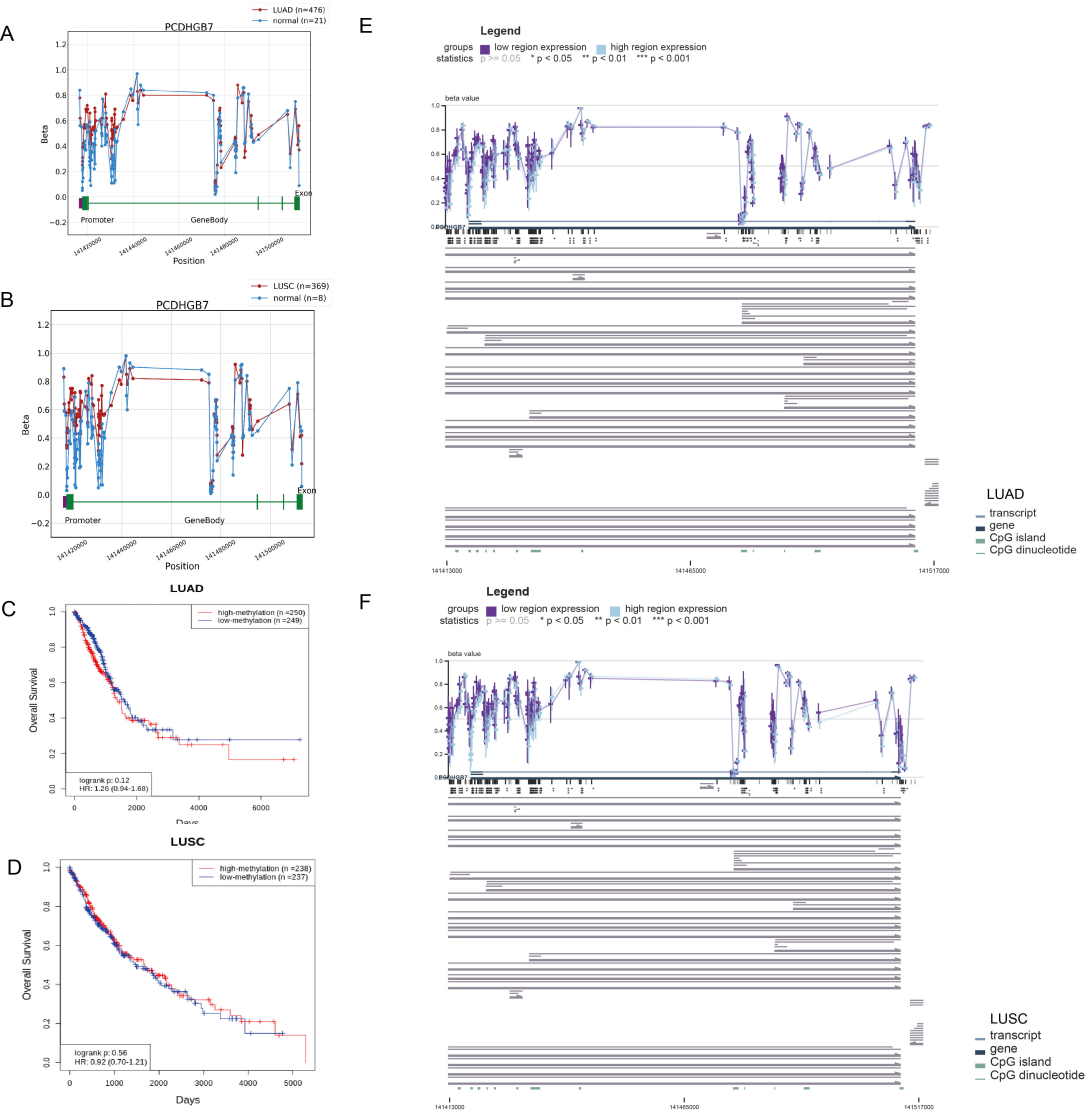
Methylation analysis of *PCDHGB7* in LUAD and LUSC. **(A)** *PCDHGB7* methylation comparison with normal tissues and survival analysis in LUAD. **(B)** *PCDHGB7* methylation comparison with normal tissues and survival analysis in LUSC. Kaplan-Meier survival analysis of *PCDHGB7* methylation in LUAD **(C)** and LUSC **(D)**. Visualization of the methylation sites within the DNA sequence associated with gene expression was performed using MEXPRESS in LUAD **(E)** and LUSC **(F)**. The expression of *PCDHGB7* is represented by the blue line. Pearson’s correlation coefficients and p-values for methylation sites and are provided on the right side. *p < 0.01, **p < 0.01, ***p < 0.001.

Dysregulated DNA methylation profiles are associated with altered gene expression in tumors (26). To investigate the relationship between aberrant *PCDHGB7* expression patterns and methylation, we used the OncoDB databases to explore abnormal *PCDHGB7* methylation patterns in normal and tumor tissues. The data revealed that the methylation levels of multiple sites in *PCDHGB7* gene were higher in tumor tissues than in normal tissues, especially in the potential promoter region (141417677 to 141419677) in both LUAD (**Figure 5A**) and LUSC (**Figure 5B**). Survival analysis revealed no significant correlation between the degree of methylation and OS in either LUAD (**Figure 5C**) or LUSC (**Figure 5D**). Additionally, we found that decreased *PCDHGB7* expression may be linked to increased methylation in LUAD (**Figure 5E**) and LUSC (**Figure 5F**) when we used MEXPRESS to investigate the relationship between *PCDHGB7* expression and CpG islands in lung cancer. The correlation coefficient and p-value of the methylation and expression correlation analysis of each DNA site are in **Supplementary Figures S3** and **S4**. These findings implied that hypermethylation of the *PCDHGB7* could contribute to the downregulated of *PCDHGB7* expression in lung cancer.

### 3.7 *PCDHGB7* methylation level in plasma is closely associated with immunotherapy efficacy

It has been reported that the level of *PCDHGB7* methylation level in malignant body fluids serves as a promising biomarker for cancer, particularly the cervical cancer (14–16). However, it remains uncertain whether the *PCDHGB7* methylation level in peripheral blood from patients with lung cancer could serve as a novel biomarker of immunotherapy efficacy. Therefore, we collected blood samples from patients with lung cancer before and after immunotherapy treatment for investigation. From October 2018 to November 2022, 32 patients were included in this study, with a median follow-up of 43.6 (30.9–62.4) months. The baseline characteristics of the enrolled patients are presented in **Supplementary Table S1**. The median age across is 65.5 years, with a slight male predominance (26 males, 82%). The pathological types are evenly distributed, with squamous cell carcinoma present in 13 patients (40.6%) and non-squamous cell carcinoma in 19 patients (59.4%). In terms of therapy, 20 patients (62.5%) received ICIs plus chemotherapy, 2 patients (6.3%) received ICIs plus chemotherapy plus anti-angiogenic therapy, 2 patients (6.3%) received immune plus anti-angiogenic therapy, and 9 patients (28.1%) received immune monotherapy. First-line therapy was administered to 24 patients (75%), and second-line and posterior line therapy to 8 patients (25%). Patients were divided into high- and low-methylation *PCDHGB7* groups based on the baseline value of plasma *PCDHGB7* methylation. Interestingly, patients with liver metastasis or a high baseline tumor load were more likely to have high plasma *PCDHGB7* methylation.

**Figure 6:**
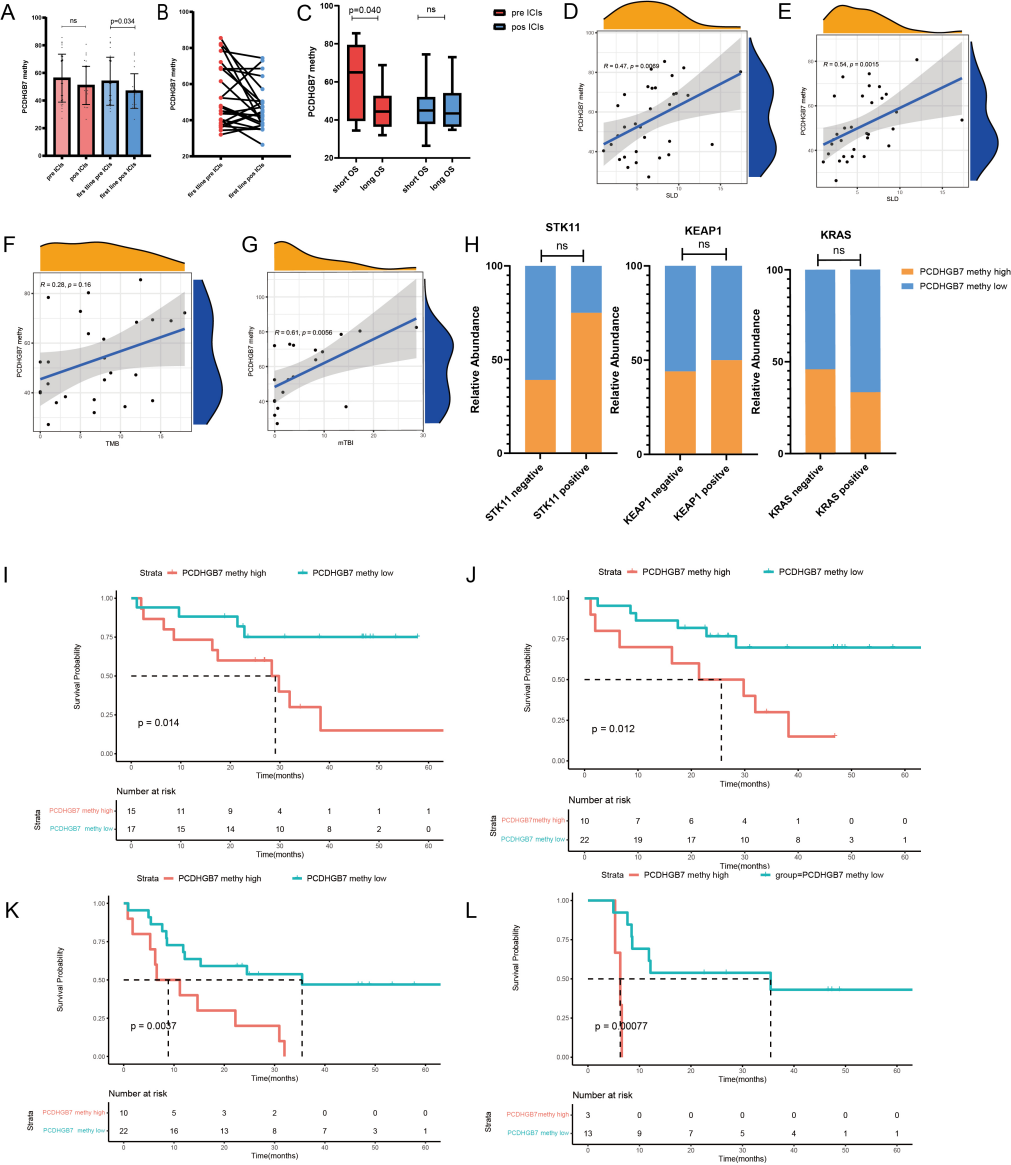
Plasma *PCDHGB7* methylation analysis of immunotherapy in lung cancer. **(A, B)** Baseline and post-treatment *PCDHGB7* methylation. **(C)** *PCDHGB7* methylation before and after treatment was grouped according to efficacy. Association between *PCDHGB7* methylation with treatment baseline SLD **(D)** and post-treatment SLD **(E)**. Association between *PCDHGB7* methylation and TMB **(F)** and mTBI **(G)**. **(H)** Analysis of *PCDHGB*7 methylation and the STK11 KEAP1 KRAS mutation. Overall survival analysis of baseline *PCDHGB*7 methylation **(I)** and post-treatment *PCDHGB*7 methylation **(J)**. Progression-free survival analysis of post-treatment *PCDHGB*7 methylation **(K)**. Progression-free survival analysis of post-treatment *PCDHGB*7 methylation in patients with SD **(L)**. ns, not significant.

Next, we explored *PCDHGB7* methylation changes in peripheral blood at baseline and early post-treatment. *PCDHGB7* methylation showed a downward trend after treatment, which was more significant in patients receiving first-line immunotherapy patients (total patients: 56.2 vs. 50.9; first line: 54.0 vs. 46.8, *p* = 0.034, **Figures 6A, B**). Besides, there was a significant correlation between the sum of longest diameters (SLD) of target lesions and *PCDHGB7* methylation, both at baseline (r = 0.47, *p* = 0.0069) and after treatment (r = 0.54, *p* = 0.0015) (**Figures 6D, E**). Patients treated with first-line immunotherapy were divided into the long OS group (> 25 months) and the short OS group (≤ 25 months) based on the OS time. The short OS group had a higher degree of methylation at baseline (60.0 vs. 45.5, *p* = 0.040, **Figure 6C**).

The association between plasma *PCDHGB7* methylation and gene mutations was equally notable, in that a statistically non-significant positive correlation (r = 0.28, *p* = 0.16) was identified between the tumor tissue TMB and plasma *PCDHGB7* methylation (**Figure 6F**). In addition, there was a strong positive correlation between *PCDHGB7* methylation and the plasma mTBI (r = 0.61, *p* = 0.0058) (**Figure 6G**). We also investigated the relationship between plasma *PCDHGB7* methylation and immune-predictive marker mutations based on the results of TCGA. The results revealed that patients with mutations of *STK11* (68.7 vs. 53.0, *p* = 0.103; χ^2^ = 1.776, *p* = 0.294) and *KEAP1* (61.2 vs. 54.9, *p* = 0.636; χ^2^ = 0.27, *p* = 1.000) tended to have higher *PCDHGB7* methylation, whereas patients with mutations of *KRAS* (55.3 vs. 55.3, *p* = 0.999; χ^2^ = 0.169, *p* = 0.869) correlated with lower P*CDHGB7* methylation (**Figure 6H**).

Survival analysis showed that plasma *PCDHGB7* methylation before treatment served as a predictive marker of immunotherapy efficacy, with patients with higher baseline plasma hypermethylation having a shorter OS (high vs. low 28.5 vs. 46.9 months, *p* = 0.014; **Figure 6I**). Patients with higher *PCDHGB7* methylation levels after treatment had a shorter OS (high vs. low (23.7 vs. 52.2 months, *p* = 0.012, **Figure 6J**) and PFS (high vs. low (13.2 vs. 39.0 months, *p* = 0.0037, **Figure 6K**), particularly those with an initial tumor evaluation of stable disease after treatment (high vs. low (6.0 vs. 37.2 months, *p <* 0.001) (**Figure 6L**).

### 3.8 Dynamic change pattern of plasma *PCDHGB7* methylation

**Figure 7:**
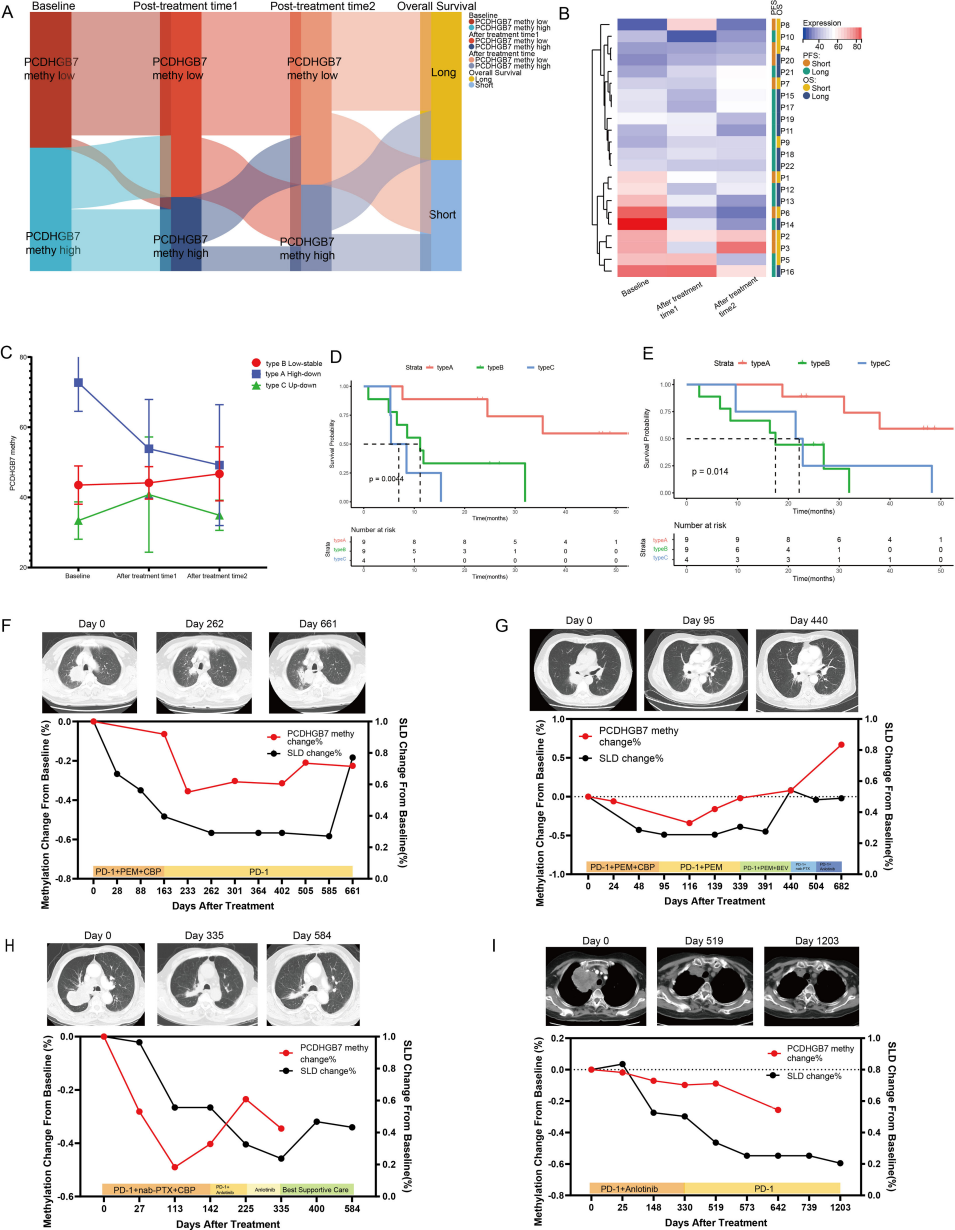
Dynamic changes in plasma *PCDHGB7* methylation. **(A)** Sankey diagram depicting *PCDHGB7* methylation at three time points and overall survival outcomes. **(B)** Heatmaps of *PCDHGB7* methylation at three time points. **(C)** Line chart of the three *PCDHGB7* methylation patterns. PFS **(D)** and OS **(E)** analyses of three methylation patterns. **(F–I)** Multipoint dynamic *PCDHGB7* methylation and SLD changes.

Twenty-one patients who received PD-1 inhibitors combined with metronomic oral vinorelbine were enrolled in our study. Peripheral blood testing for *PCDHGB7* methylation was conducted, and patients were divided into three groups: treatment-naive, 1–2 stages after treatment (post-treatment time point 1), and 3–4 stages after treatment (post-treatment time point 2). A Sankey diagram was used to show changes in methylation levels and OS in each patient at these three time points. The results indicated that patients with consistently high methylation throughout the study period and after treatment had shorter OS, whereas those with consistently low methylation had longer OS (**Figure 7A**). We created heatmaps of *PCDHGB7* methylation levels at three time points (**Figure 7B**), and the methylation patterns of patients were divided into three categories based on the clustering results. The line chart shows the methylation changes in three modes: type A (high-down: the baseline methylation value of treatment was relatively high and continuously decreased with immunotherapy), type B (low-stable: both the baseline and post-treatment methylation levels remained low), and type C (up-down: the initial methylation level was low but increased and then decreased during the treatment process) (**Figure 7C**). PFS and OS analyses of these three metal patterns showed that patients with type A pattern had significantly better prognosis than those with type B and type C patterns, suggesting that dynamic monitoring of plasma methylation can effectively predict the efficacy of immunotherapy (**Figures 7D, E**) (type A, NA, 95% CI [24.50–NA]; type B, 11.17 months, 95% CI [6.57–NA]; type C, 6.93 months, 95% CI [5.27–NA] [p = 0.0044]; and OS, type A, NA, 95% CI [30.97–NA]; type B, 17.5 months, 95% CI [8.6–NA]; type C, 22.1 months, 95% CI [9.63–NA]; p = 0.014).

Finally, we performed dynamic, all-encompassing multi-timepoint surveillance of a subset of patients who continued to receive immunotherapy. Analysis was performed on changes in SLDs on computed tomography (CT) scans. The patients depicted in **Figures 7F–H** initially received a combination of immunotherapy and chemotherapy, while their front-line treatment subsequently transitioned to a changed chemotherapy regimen or immunotherapy plus anti-angiogenic therapy following disease progression. In these three patients, a tendency toward declining to the lowest point and then increasing was observed for both *PCDHGB7* methylation and SLDs. Notably, peripheral blood *PCDHGB7* methylation events preceded the radiological recurrence of tumors on CT images by approximately 101–175 days, suggesting a potential epigenetic prelude to clinical relapse. The patient shown in **Figure 7I** received immunotherapy in addition to anti-angiogenic medication, and prolonged disease control was achieved for approximately 3 years, as evidenced by a steady decrease in plasma *PCDHGB7* methylation levels.

### 3.9 Plasma *PCDHGB7* protein level is a prognostic biomarker for lung cancer immunotherapy

We further analyzed plasma *PCDHGB7* levels in a prospective clinical cohort of elderly patients with non-small cell lung cancer. Patients were treated with a PD-1 inhibitor combined with metagenomic oral vinorelbine as a posterior line therapy. The baseline clinical characteristics of the ten patients are presented in **Supplementary Table S2**. There are a total of 10 patients with a majority being male (70%) and a median age of 68 years. The majority of patients have an ECOG performance status of 0 (60%). Almost all patients are in TNM Stage IV (90%), with the pathological type split between non-squamous cell carcinoma (60%) and squamous cell carcinoma (40%). Metastases to the brain are present in 10% of the patients.

**Figure 8:**
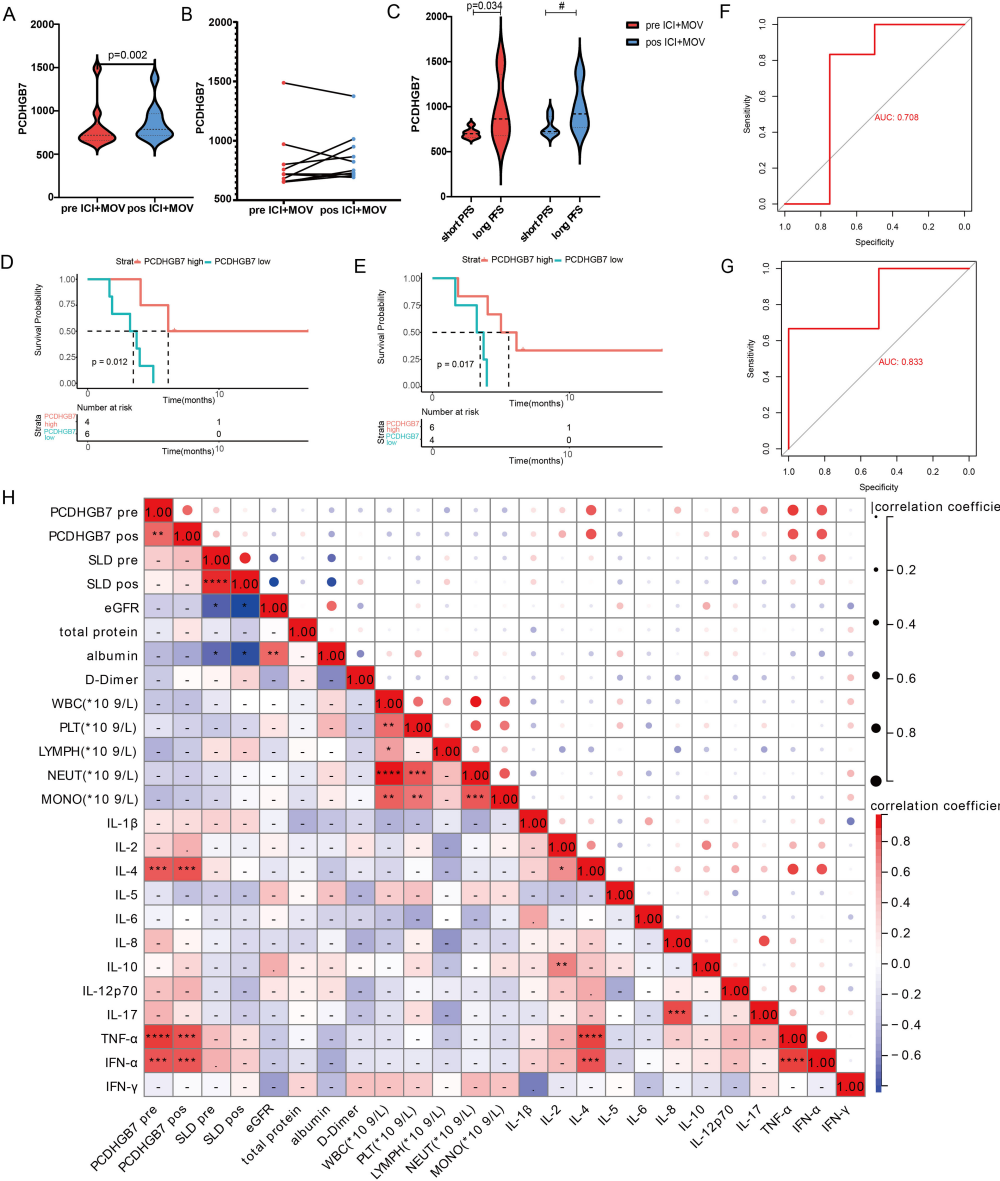
Analysis of baseline and post-treatment plasma PCDHGB7 protein levels. **(A, B)** Baseline and post-treatment plasma PCDHGB7 protein level. **(C)** Plasma PCDHGB7 before and after treatment was grouped according to efficacy. PFS analysis of baseline plasma PCDHGB7 **(D)** and post-treatment plasma PCDHGB7 **(E)**. ROC curve analysis of baseline plasma PCDHGB7 **(F)** and post-treatment plasma PCDHGB7 **(G)**. **(H)** Correlation matrix between plasma PCDHGB7 and various clinical indicators (*p< 0.05, **p< 0.01, ***p < 0.001, ****p < 0.0001). ns, not significant.

A significant increase in the plasma *PCDHGB7* protein level was observed after treatment (810.0 *vs.* 863, p = 0.002, **Figures 8A, B**). Patients were further divided into two groups based on PFS. Patients in the long PFS group (≥ 5 months) had a higher degree of *PCDHGB7* at baseline (708 *vs.* 1074, p = 0.034, **Figure 8C**). We also analyzed the relationship between *PCDHGB7* protein expression and PFS at baseline and after treatment. The results showed that patients with higher levels of plasma *PCDHGB7* protein had a longer PFS at baseline (*PCDHGB7* high: 6.13 months, 95% CI [4.03–NA], *PCDHGB7* low: 3.48 months, 95% CI [1.87–NA]) and after treatment (*PCDHGB7* high: 5.57 months, 95% CI [4.03–NA], *PCDHGB7* low: 3.48 months, 95% CI [1.67–NA]) (**Figures 8D, E**). According to the ROC curve analysis, the plasma protein level was a promising indicator of the efficacy of immunotherapy (**Figures 8F, G**).

Further analysis was performed to examine the correlation between plasma *PCDHGB7* protein levels and peripheral blood immune cells and cytokines, as well as various clinical parameters. Remarkably, *PCDHGB7* protein levels exhibited significant positive associations with interleukin-4 (IL-4, p < 0.0001), tumor necrosis factor-alpha (TNF-α, p < 0.0001), and interferon-alpha (IFN-α, p < 0.001) (**Figure 8H**).

## 4 Discussion

PCDHs are a group of transmembrane proteins belonging to the cadherin superfamily and have been proven to play major tumor suppressor functions by inhibiting the proliferation and metastasis of cancer cells (6, 10). Unlike other members of the PCDHs family, the association between *PCDHGB7* and cancer has not been fully understood. Few reports have been made to claim *PCDHGB7* could be a novel marker for breast cancer, non-Hodgkin’s lymphoma, and hepatocellular carcinoma (27–29). Recently, *PCDHGB7* methylation was identified as a diagnosis indicator for cervical cancer, endometrial cancer, and malignant body fluids which attracted more oncologists’ attention since its huge translational potential (14–16, 30). According to our comprehensive analysis, *PCDHGB7* expression was downregulated in LUAD and LUSC and associated with tumor prognosis. *PCDHGB7* was found to demonstrate a positive correlation with inhibitory immune cells and a negative correlation with TMB and HRD. The data suggested that *PCDHGB7* may play an important role in tumor initiation and development by affecting the immune response and DNA damage in tumor microenvironment.

Similar to other investigations, we also found high expression of *PCDHGB7* in lung cancer (14), we further deciphered the role of *PCDHGB7* in lung cancer that has not yet been fully explored yet. Low gene expression is thought to have been caused by methylation of the DNA promoter region (31). Utilizing the OncoDB and MEXPRESS databases, we examined the methylation status of the *PCDHGB7* gene in lung cancer and discovered a negative correlation between the methylation level and *PCDHGB7* expression at most gene sites, particularly the promoter region (2 KB upstream of the gene). According to earlier research using public database analysis, over 80% of breast tumors display aberrant methylation of the *PCDHGB7* gene. Meanwhile, the detection of *PCDHGB7* mRNA expression in breast tissue and breast cancer cell lines e indicated that *PCDHGB7* expression was downregulated in breast cancer tissue due to hypermethylation of the promoter region (32). Nevertheless, further immunohistochemical and lung cancer cell line detection data are required to ascertain the connection between *PCDHGB7* promoter methylation and expression.

Although multiple data from TCGA LUSC and GEO databases indicate that high expression of *PCDHGB7* is associated with poor prognosis, there is a potential contradiction in the prognostic role of *PCDHGB7* in lung squamous cell carcinoma and adenocarcinoma, which requires further validation with larger sample sizes in the future. We believe that these potential differences may be due to different biological behaviors and molecular pathways related to LUAD and LUSC. Especially with the development of single-cell technology, studies have identified significant differences in the immune microenvironmental signals between LUAD and LUSC (33). In this study, *PCHDGB7* showed different mutation landscapes in lung adenocarcinoma and lung squamous cell carcinoma, and the analysis of immune infiltrating cells was not the same, which may lead to its different prognostic value in the two types of lung cancer.

DNA mismatch repair (MMR) is an important DNA repair pathway that plays a critical role in DNA replication fidelity, mutation avoidance, and genome stability. A hypermutated phenotype in the genome caused by MMR deficiency ultimately results in MSI (34). Specifically, MMR-deficient cancers tend to be more sensitive to immune checkpoint blockade (35). Meanwhile, research continues on the HRD score as a biomarker of response to various therapies in non-small cell lung cancer. In our study, HRD analysis revealed *PCDHGB7* expression showed a negative correlation with HRD in LUAD. According to the protein interaction network, *PCDHGB7* shares a close relationship with proteins such as FAT1, MLH1, and the FHIT. Patients with LUSC who have low expression of *PCDHGB7* are more likely to develop FAT1 mutations, while GSEA analysis revealed that high expression of PCDHGB was linked to the activation of homologous recombination, or mismatch repair, in LUAD (**Figure 4F**). These comprehensive investigations suggest that *PCDHGB7* might play a significant role in lung cancer DNA mismatch repair. Doissy et al. demonstrated an exceptional duration of response of more than 20 months in a patient with LUSC who received platinum-based therapy and had a high HRD score (36). Although the specific mechanism by which *PCDHGB7* participates in DNA MMR requires further exploration, the clinical significance of this phenomenon will grow with the extensive investigation into DNA damage repair inhibitors, such as poly-ADP ribose polymerase inhibitors, in patients with NSCLC (37).

Since the revolution of the new treatment paradigm of immunotherapy for lung cancer, the urgent need for novel predictive markers of the efficacy of immunotherapy has dramatically increased (38). The data from the IMvigor210 cohort and Kim cohort showed that patients with high expression of PCDHGB7 in tumor tissue exhibited significantly poor responses to immunotherapy, which may be due to its negative correlation with TMB, as well as its significant correlation with M2 macrophages and Treg cells. TMB is a widely used positive predictive indicator for the efficacy of immunotherapy in clinical practice (39). Numerous studies have shown that M2 macrophages play an important role in mediating tumor invasion and metastasis, immune suppression, and treatment resistance (40, 41). Infiltrating regulatory T cells (Tregs) are also important contributors to immunosuppressive and key targets for cancer immunotherapy (42). Meanwhile, we investigated the relationship between plasma *PCDHGB7* methylation and immunotherapy response in patients with NSCLC who received immunotherapy at our center. Patients with baseline plasma hypermethylation had a shorter PFS and OS, while their *PCDHGB7* methylation levels decreased after treatment. A specific clinical reason for the predictive diagnostic function of *PCDHGB7* methylation can be found in its association with ctDNA, mTBI, and tumor burden. Furthermore, using methylation detection at baseline and two treatment time periods, we attempted to derive the *PCDHGB7* methylation alteration pattern. Similar to the monitoring mode for the continuous clearance of ctDNA, the results indicated that patients with early reduction in DNA methylation had the best prognosis (43, 44). Combined with the fact that the methylation results may reveal the disease progression of patients in advance of imaging, *PCDHGB7* methylation monitoring shows better stability and health economics effects than ctDNA and exosome detection; moreover, because of its noninvasive multi-point dynamic detection, prospective studies with larger samples can be conducted in the future to verify the above results.

Finally, our data showed that patients with higher plasma *PCDHGB7* levels had better outcomes of immune checkpoint inhibitor treatment, and both baseline and post-treatment protein levels had good diagnostic efficacy for PFS. Notably, the *PCDHGB7* protein is associated with two common inflammatory stimuli, IL-4 and TNF-α. IL-4 plays a critical role in the humoral immune response, antibody generation, and T and B cell development (45), while recent studies have suggested that TNF-α may participate in the killing effect of immunotherapy against tumors (46). Furthermore, our ensemble pathway enrichment analysis indicated that *PCDHGB7* may affect the efficacy of lung cancer immunotherapy by regulating the interaction between cytokines and their paired receptors.

In summary, our research findings indicate that increased methylation of *PCHDGB7* in lung adenocarcinoma and lung squamous cell carcinoma tissues leads to decreased expression compared to normal tissues. In lung cancer tissues, high expression of *PCDHGB7* is associated with multiple pathways, such as homologous recombination, DNA mismatch repair, and JAK-stat pathway, while significantly increasing the infiltration of immune system negatively regulated cells. Therefore, high expression of *PCDHGB7* may lead to disorders in DNA damage repair and anti-tumor immune regulation, resulting in poor responsiveness of lung cancer patients to immunotherapy. We further hypothesize that the methylation status of *PCDHGB7* in ctDNA can reflect the overall epigenetic state of the tumor, and this state has a more accurate predictive value for the efficacy of immunotherapy. The dynamic changes in methylation and expression levels of plasma *PCDHGB7* can serve as biomarkers for immunotherapy of lung cancer.

While our study provides preliminary insights into the potential of *PCDHGB7* as a predictive biomarker for immunotherapy, the limitations underscore the need for further research. Our ability to explore the predictive role of *PCDHGB7* in immunotherapy was constrained by the availability of tumor tissue for *PCDHGB7* detection. This constraint prevented us from elucidating potential contradictions in the predictive role of *PCDHGB7* between tumor tissue and peripheral blood. Secondly, the insufficient sample size of the single clinical center limits the generalizability of the results drawn in this study. In addition to the clinical limitations, there is a pressing need for further cellular and animal studies to explore the biological functions of *PCDHGB7* in lung cancer. Addressing these limitations through larger, multicenter studies and deeper biological investigations will be instrumental in advancing our understanding of *PCHDGB7*’s role in immunotherapy and its potential as a clinical tool for personalized treatment strategies.

## 5 Conclusion

Our data illustrated that *PCDHGB7* expression and methylation are prognostic and immunological biomarkers in non-small cell lung cancer. Plasma *PCDHGB7* methylation and protein levels can be used as novel biomarkers for predicting the efficacy of immunotherapy against lung cancer.

## Data Availability

The original contributions presented in the study are included in the article/**Supplementary Material**. Some datasets presented in this article are not readily available due to the ethical considerations and intellectual property regulations that govern the sharing of clinical data and plasma assay data. Requests to access these datasets should be directed to the corresponding author.
