# Supplementary material for: Deregulated methylation and expression of PCDHGB7 in patients with non-small cell lung cancer: a novel prognostic and immunological biomarker
Source: Front Immunol. 2025 Jan 30;16:1516628. doi: 10.3389/fimmu.2025.1516628 (PMC11821955; doi:10.3389/fimmu.2025.1516628)
Supplement: Supplementary file 1 [file Table1.docx]

Supplemental Table S1: The baseline characteristics of the plasma *PCDHGB7* methylation detection cohorts.

| Characteristic | High, n = 15 | Low, n = 17 | P-value |
| --- | --- | --- | --- |
| Age (y) | 65 (62, 68) | 66 (60, 70) | 0.6 |
| Sex |  |  | > 0.9 |
| Male | 13 (87%) | 14 (82%) |  |
| Female | 2 (13%) | 3 (18%) |  |
| Pathological type | |  | > 0.9 |
| Squamous cell carcinoma | 6 (40%) | 7 (41%) |  |
| Non-squamous cell carcinoma | 9 (60%) | 10 (59%) |  |
| TNM Stage |  |  | 0.5 |
| III | 6 (40%) | 4 (24%) |  |
| IV | 9 (60%) | 13 (76%) |  |
| TMB |  |  | 0.2 |
| H | 4 (27%) | 3 (18%) |  |
| L | 6 (40%) | 12 (71%) |  |
| NA | 5 (33%) | 2 (12%) |  |
| PD-L1 |  |  | 0.9 |
| < 1% | 5 (33%) | 5 (29%) |  |
| 1%–49% | 3 (20%) | 4 (24%) |  |
| ≥ 50% | 3 (20%) | 3 (18%) |  |
| NA | 4 (27%) | 5 (29%) |  |
| Brain metastases |  |  | 0.3 |
| No | 12 (80%) | 16 (94%) |  |
| Yes | 3 (20%) | 1 (5.9%) |  |
| Bone metastases |  |  | > 0.9 |
| No | 10 (67%) | 11 (65%) |  |
| Yes | 5 (33%) | 6 (35%) |  |
| Liver metastases |  |  | 0.038 |
| No | 11 (73%) | 17 (100%) |  |
| Yes | 4 (27%) | 0 (0%) |  |
| Size of largest tumor diameter | 8.3 (6.7, 9.6) | 4.9 (3.5, 6.4) | 0.008 |
| Therapy |  |  | 0.8 |
| ICIs + chemotherapy | 8 (53%) | 12 (71%) |  |
| ICIs + chemotherapy + anti-angiogenic | 1 (6.7%) | 0 (0%) |  |
| Immune + anti-angiogenic | 1 (6.7%) | 1 (5.9%) |  |
| Immune monotherapy | 5 (33%) | 4 (24%) |  |
| ICIs line |  |  |  |
| First line | 9 (60%) | 15 (88%) | 0.2 |
| Second line and posterior line | 6 (40%) | 2 (12%) |  |

TNM:Tumor-Node-Metastasis. TMB: Tumor mutation burden. ICI: immune checkpoint inhibitor.
