# Supplementary material for: Deregulated methylation and expression of PCDHGB7 in patients with non-small cell lung cancer: a novel prognostic and immunological biomarker
Source: Front Immunol. 2025 Jan 30;16:1516628. doi: 10.3389/fimmu.2025.1516628 (PMC11821955; doi:10.3389/fimmu.2025.1516628)
Supplement: Supplementary file 2 [file Table2.docx]

Supplemental Table S2: The baseline characteristics of the plasma PCDHGB7protein level detection cohorts.

| Characteristic | High, n = 4 | Low, n = 6 | P-value |
| --- | --- | --- | --- |
| Sex |  |  | > 0.9 |
| Female | 1 (25%) | 2 (33%) |  |
| Male | 3 (75%) | 4 (67%) |  |
| Age (y) | 68 (65, 73) | 67 (65, 70) | 0.6 |
| ECOG |  |  | 0.7 |
| 0 | 2 (50%) | 4 (67%) |  |
| 1 | 2 (50%) | 1 (17%) |  |
| 3 | 0 (0%) | 1 (17%) |  |
| Smoking history |  |  | 0.5 |
| 0 | 2 (50%) | 2 (33%) |  |
| 0–200 | 0 (0%) | 2 (33%) |  |
| 200–400 | 2 (50%) | 2 (33%) |  |
| Charlson Comorbidity Index (CCI) | |  | 0.2 |
| 1 | 0 (0%) | 2 (33%) |  |
| 3 | 3 (75%) | 0 (0%) |  |
| 4 | 1 (25%) | 2 (33%) |  |
| 5 | 0 (0%) | 1 (17%) |  |
| 6 | 0 (0%) | 1 (17%) |  |
| TNM stage |  |  | > 0.9 |
| Stage III | 0 (0%) | 1 (17%) |  |
| Stage IV | 4 (100%) | 5 (83%) |  |
| Pathological type |  |  | 0.6 |
| Non-squamous cell carcinoma | 3 (75%) | 3 (50%) |  |
| Squamous cell carcinoma | 1 (25%) | 3 (50%) |  |
| Brain metastases | 1 (25%) | 0 (0%) | 0.4 |
| Liver metastases | 1 (25%) | 2 (33%) | > 0.9 |
| Bone metastases | 2 (50%) | 3 (50%) | > 0.9 |
| PD-L1 |  |  | > 0.9 |
| < 1% | 2(50%) | 3 (51%) |  |
| 1%–49% | 1 (25%) | 1 (17%) |  |
| ≥ 50% | 0 (0%) | 1 (17%) |  |
| NA | 1 (25%) | 1 (17%) |  |
| TMB |  |  | 0.4 |
| H | 1 (25%) | 3 (50%) |  |
| L | 0 (0%) | 2 (33%) |  |
| NA | 3 (75%) | 1 (17%) |  |

ECOG PS: Eastern Cooperative Oncology Group Performance Status. TNM stage: Tumor-Node-Metastasis. TMB: Tumor mutation burden.
