# Supplementary material for: Deregulated methylation and expression of PCDHGB7 in patients with non-small cell lung cancer: a novel prognostic and immunological biomarker
Source: Front Immunol. 2025 Jan 30;16:1516628. doi: 10.3389/fimmu.2025.1516628 (PMC11821955; doi:10.3389/fimmu.2025.1516628)
Supplement: Supplementary file 3 [file DataSheet1.pdf]

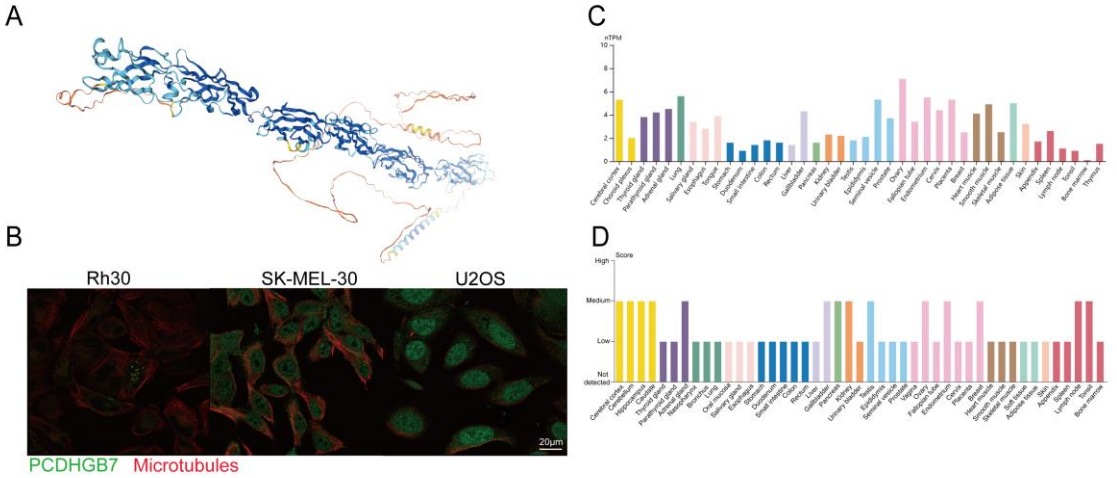

Supplementary Figure S1. Overview of PCDHGB7. (A)Representation of the molecular structure of PCDHGB7. (B) Visualization of the subcellular distribution of PCDHGB7 within cells. (C) Analysis of PCDHGB7 expression at the RNA level across diverse organ types, providing insights into the transcriptomic landscape. (D) Distribution of PCDHGB7 expression at the protein level.
