# Supplementary material for: Deregulated methylation and expression of PCDHGB7 in patients with non-small cell lung cancer: a novel prognostic and immunological biomarker
Source: Front Immunol. 2025 Jan 30;16:1516628. doi: 10.3389/fimmu.2025.1516628 (PMC11821955; doi:10.3389/fimmu.2025.1516628)
Supplement: Supplementary file 4 [file DataSheet2.pdf]

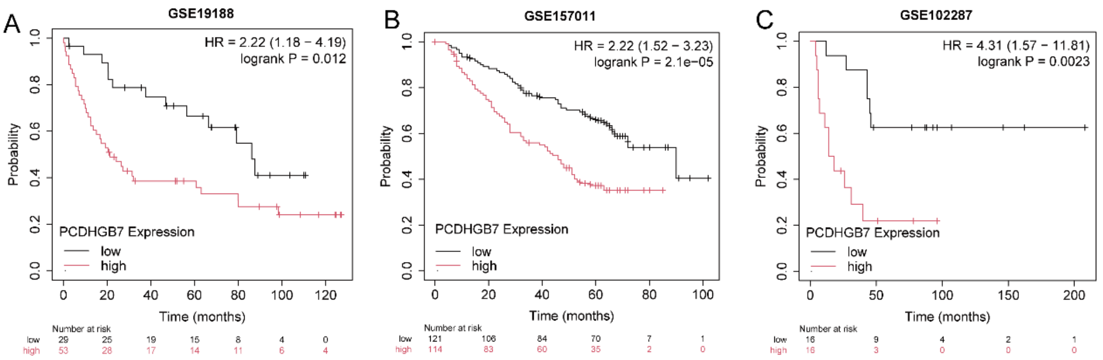

Supplementary Figure S2. Prognostic potential of *PCDHGB7* in lung cancer. (A) Kaplan-Meier survival analysis of GSE19188(expression data of early-stage non-small cell lung cancer). (B) Kaplan-Meier survival analysis of GSE157011(expression data of early-stage squamous lung cancer). (C) Kaplan-Meier survival analysis of GSE102287(gene data from African Americans and European Americans with non-small cell lung cancer).
