# Supplementary material for: Deregulated methylation and expression of PCDHGB7 in patients with non-small cell lung cancer: a novel prognostic and immunological biomarker
Source: Front Immunol. 2025 Jan 30;16:1516628. doi: 10.3389/fimmu.2025.1516628 (PMC11821955; doi:10.3389/fimmu.2025.1516628)
Supplement: Supplementary file 5 [file DataSheet3.pdf]

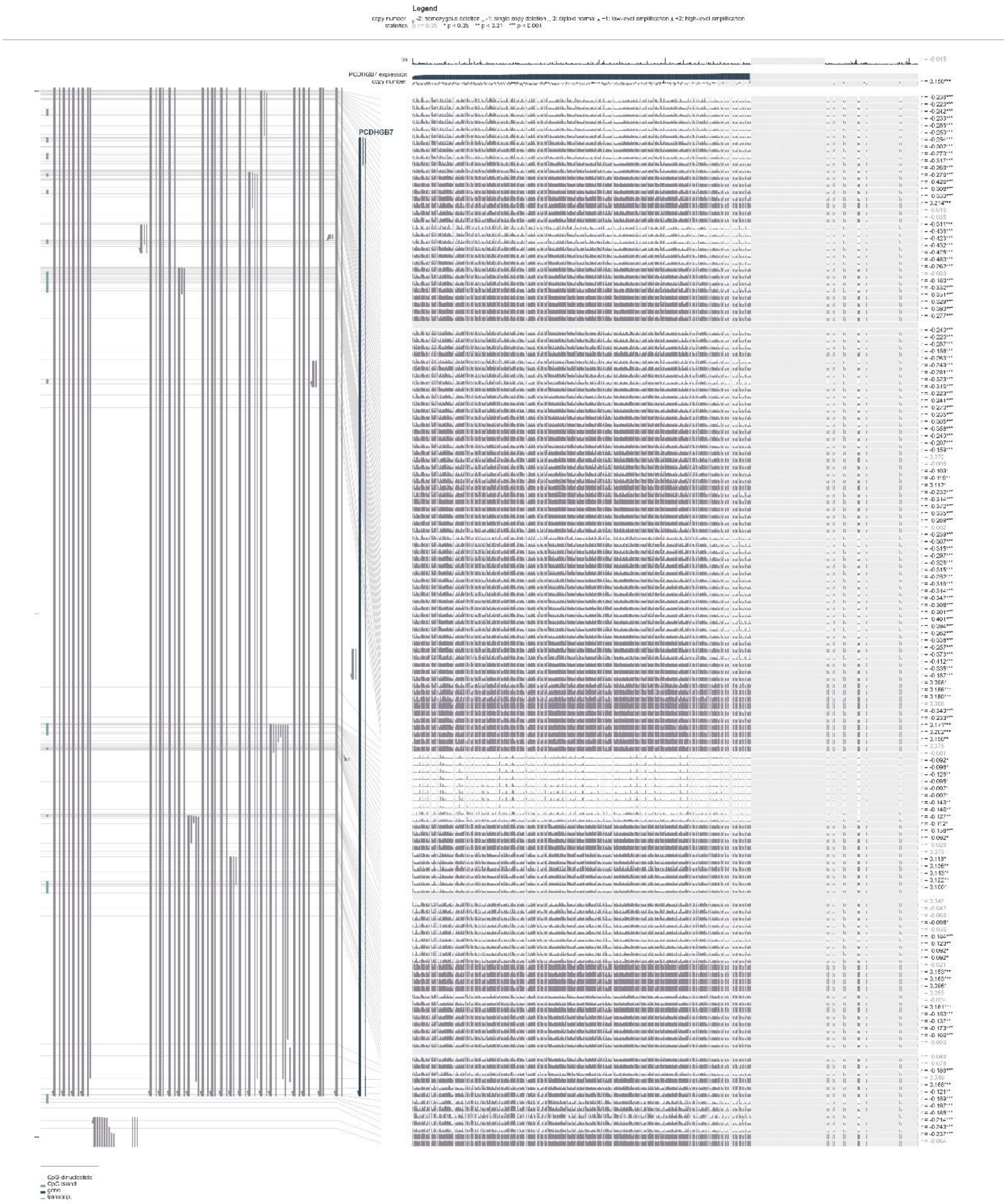

Supplementary Figure S3. Visualization of DNA methylation and mRNA expression data for *PCDHGB7* in patients with lung adenocarcinoma analyzed by MEXPRESS. The significance of the relation (correlation coefficient r or *p* value) between expression and methylation was shown in the right side. *p<0.05; **p<0.01; ***p<0.001.
